# Current status of treatment of cancer-associated venous thromboembolism

**DOI:** 10.1186/s12959-021-00274-x

**Published:** 2021-03-31

**Authors:** Wei Xiong

**Affiliations:** Department of Pulmonary and Critical Care Medicine, Xinhua Hospital, Affiliated to Shanghai Jiaotong University School of Medicine, No. 1665, Kongjiang Road, Yangpu District, Shanghai, 200092 China

**Keywords:** Cancer, Venous thromboembolism, Treatment, Current status, Anticoagulation

## Abstract

Patients with cancer are prone to develop venous thromboembolism (VTE) that is the second leading cause of mortality among them. Cancer patients with VTE may encounter higher rates of VTE recurrence and bleeding complications than patients without cancer. Treatment of established VTE is often complex in patients with cancer. Treatment of cancer-associated VTE basically comprises initial treatment, long-term treatment, treatment within 6 months, treatment beyond 6 months, treatment of recurrent VTE, and treatment in special situations. Decision of antithrombotic therapy, selection of anticoagulants, duration of anticoagulation, decision of adjuvant therapy, and adjustment of regimen in special situations are the major problems in the treatment of cancer-associated VTE. Therapeutic anticoagulation is the key of the key in the treatment of cancer-associated VTE. In addition to the efficacy and safety of low-molecular-weight heparin (LMWH) that has been fully demonstrated, direct oral anticoagulants (DOACs) are increasingly showing its advantages along with the accompanying concern in the treatment of cancer-associated VTE. The latest ASCO, ITAC and NCCN guidelines agree with each other on most aspects with respect to the treatment of cancer-associated VTE, whereas differ on a few issues. Encompassing recent randomized controlled trials, clinical trials, and meta-analyses, as well as the comparison of the latest authoritative guidelines including the NCCN, ASCO, and ITAC guidelines in this field, the objective of this review is to present current overview and recommendations for the treatment of cancer-associated VTE.

## Introduction

Cancer-associated venous thromboembolism (VTE) is a common and life-threatening condition in adult patients (≥18 years) with cancer [1]. Patients with cancer are four to seven times more likely to develop VTE than patients without cancer [2]. The factors that are responsible for the increase of cancer-associated VTE incidence basically include cancer type and anticancer-associated treatment [2, 3]. The statistical data showed that VTE was observed in 8.4% of nearly six million hospitalizations of 3,146,388 individual patients with cancer in the United States over a nearly two-decade period [4]. The incidence of cancer-associated VTE is still increasing worldwide [2].

Cancer-associated VTE is an important cause of morbidity for patients with cancer [5]. Patients with cancer may require critical care for the complication of their malignancy. Pulmonary embolism is one of the complications that can lead to the intensive care unit (ICU) admission for patients with cancer [6]. VTE is the second leading cause of mortality among patients with cancer after disease progression [2]. A large scale survey revealed that in-hospital mortality was observed in 5.5% of cancer patients without a VTE diagnosis, whereas in 15.0% of those with VTE, including 19.4% with a pulmonary embolism, in the United States [4].

For established cancer-associated VTE, the classifications of treatments currently comprise initial treatment, long-term treatment, treatment within 6 months, treatment beyond 6 months, treatment of catheter-related VTE, treatment of recurrent VTE, and treatment in special situations. Current therapeutic agents of cancer-associated VTE mainly comprise low-molecular-weight heparin (LMWH), direct oral anticoagulants (DOACs), unfractionated heparin (UFH), fondaparinux, vitamin K antagonist (VKA), and inferior vena cava filter (IVCF) [1, 2, 5].

Nevertheless, the treatment of established VTE in cancer patients is complicated [2]. Patients with cancer may encounter higher rates of VTE recurrence and bleeding complications than patients without cancer [5]. In addition to the efficacy and safety of LMWH that has been acknowledged, DOACs have demonstrated a favorable risk–benefit ratio and advantages over parenteral anticoagulants, meanwhile, they also pose challenges with respect to oral administration, drug–drug interactions, and bleeding risk [2]. Comprehensive treatment of cancer-associated VTE should be focused on the identification of patients who will mostly benefit from the treatment to reduce its recurrence and mortality [5]. Decision of antithrombotic therapy, selection of anticoagulants, duration of anticoagulation, decision of adjuvant therapy, and adjustment of regimen in special situations constitute the major problems in the treatment of cancer-associated VTE.

## Overview

To date, the pharmacological anticoagulant agents most frequently used for cancer-associated VTE are LMWH and DOACs. In a real-world analysis, from September 2018 to January 2020, LMWH was the most common initial anticoagulation treatment for VTE in patients with cancer. Nevertheless, DOACs had a lower risk of recurrent venous thromboembolism compared with LMWH or warfarin [7]. In a retrospective cohort study, LMWH and UFH were the most common initial treatments (35.2 and 27.4%, respectively) of cancer-associated VTE, followed by DOACs (9.6%). Most of patients (71.4%) who received DOACs sticked to the post-discharge DOACs medication, whereas only 24.1, 43.5, and 0.1% of patients receiving LMWH, warfarin, and UFH remained on the same anticoagulant after discharge, respectively. DOACs were the most common initial post-discharge outpatient option. Persistence and adherence in outpatients appeared higher in patients using DOACs or warfarin versus LMWH or UFH [8]. In another retrospective cohort study, from 2000 through 2003, warfarin ± injectable was used in approximately 90% of cases. After 2003, there was a steady decline in warfarin use (90% in 2003 to 25% in 2017) corresponding with an increase in LMWH use (11% in 2003 to 55% in 2017). The regimen of DOACs ± injectable has rapidly increased from < 1% in 2014 to 20% in 2017 [9]. In two recent meta-analyses, recurrent VTE incidence was significantly decreased with DOACs compared to LMWH [10, 11], with comparable [10] or increased [11] bleeding risks. VTE recurrence was also decreased with DOACs compared to VKA, with comparable [10] or decreased [11] bleeding risks. LMWH was associated with significantly reduced VTE recurrence compared with VKA [11].

## Treatment of established Cancer-associated VTE within 6 months

Treatment of established cancer-associated VTE within 6 months consists of initial treatment (first 5–10 days) and early maintenance (up to 6 months) in the International Initiative on Thrombosis and Cancer (ITAC) guidelines [2], whereas of initial treatment (first 5–10 days) and long-term treatment (up to 6 months) in the American Society of Clinical Oncology (ASCO) guidelines [5]. Initial treatment of cancer-associated VTE is often defined as acute treatment especially the first 5 to 10 days of anticoagulant treatment [1, 2, 5, 12]. In the 2018 Cochrane database of systematic reviews with respect to the initial treatment of cancer-associated VTE, LMWH was possibly superior to adjusted-dose UFH [12].

With respect to the frequency of usage of LMWH, in a retrospective study, clinically relevant bleeding (CRB) was higher in the twice-daily enoxaparin group compared with the once-daily group at 30 days (5.3% vs 2.4%, *P* = 0.587). The composite outcome of CRB, VTE, and mortality rates was higher in the once-daily enoxaparin group than twice-daily group at all time points [13].

The recent Caravaggio trial assessed the efficacy and safety of apixaban in 1155 patients with cancer who had symptomatic or incidental acute VTE. Recurrent VTE occurred in 32 of 576 patients (5.6%) in the apixaban group and in 46 of 579 patients (7.9%) in the dalteparin group (HR, 0.63; 95% CI, 0.37 to 1.07; *P* < 0.001 for noninferiority). Major bleeding events occurred in 22 patients (3.8%) in the apixaban group and in 23 patients (4.0%) in the dalteparin group (hazard ratio, 0.82; 95% CI, 0.40 to 1.69; *P* = 0.60) [14].

In a recent meta-analysis of initial treatment of cancer-associated VTE, DOACs resulted in a lower incidence of 6-month recurrent VTE in comparison with LMWH (RR 0.56, 95% CI 0.40–0.79; *p* < 0.001). Incidence of major bleeding (RR 1.56, 95% CI 0.95–2.47, *p* = n.s.) and mortality rates (RR 1.03, 95% CI 0.91–2.47, *p* = n.s.) were comparable between DOACs and LMWH groups [15]. In another meta-analysis comparing apixaban, edoxaban, or rivaroxaban and dalteparin, recurrent VTE occurred in 75 of 1446 patients (5.2%) receiving oral factor Xa inhibitors and in 119 of 1448 patients (8.2%) receiving LMWH (RR 0.62; 95% CI 0.43–0.91; I^2^, 30%). Major bleeding occurred in 62 (4.3%) patients receiving oral factor Xa inhibitors and 48 (3.3%) patients receiving LMWH, respectively (RR 1.31; 95% CI 0.83–2.08; I^2^, 23%) [16].

Besides pharmacological anticoagulant agents, mechanotherapy may play a role in the case of contraindications to pharmacological anticoagulation. With respect to IVCF, a cohort study demonstrated that among 33, 740 cancer patients with acute lower extremity DVT and bleeding risk factors who underwent IVCF placement, 4492 patients (5.1%) developed a new PE after the initial DVT diagnosis. A significant improvement was observed in PE-free survival for patients who received IVCF compared with those who did not [17]. In another cohort study, a significant lower risk of PE-related mortality (0.8% vs 4.0%; *p* = 0.04) were observed in patients receiving IVCF due to contraindication against anticoagulation compared with those who did not, without significant between-group difference regarding the major bleeding rate (6.1% vs 5.7%; *p* = 0.85). However, recurrent VTE rates were higher in patients who received IVCF compared with those who did not (7.3% vs 3.2%; *p* = 0.05) [18].

The evidence regarding thrombolysis for cancer-associated VTE are scarce. In an observational study, no significant difference was observed regarding the in-hospital mortality between 1287 cancer patients with proximal DVT undergoing catheter-directed thrombolysis (CDT) plus anticoagulation and 1287 ones treated with anticoagulation alone (2.6% vs 1.9%; *P* = 0.23). Furthermore, CDT was associated with higher risk of intracranial hemorrhage (1.3% vs 0.4%; *P* = 0.017), blood transfusion rate (18.6% vs 13.1%; *P* < 0.001), and hematoma rate (2.4%vs 0.4%; *P* < 0.001), compared with no CDT [19].

## Treatment of established Cancer-associated VTE beyond 6 months

Treatment of established cancer-associated VTE beyond 6 months denotes long-term treatment in the ITAC guidelines [2], whereas treatment after 6 months in the ASCO guidelines [5]. The anticoagulation beyond 6 months often depends on a case-by-case basis. The 2018 Cochrane database of systematic reviews with respect to the long-term treatment of cancer-associated VTE showed that LMWH probably resulted in an important reduction in VTE compared with VKA. DOACs may likely reduce VTE compared with LMWH, but may increase major bleeding risk [20].

The Hokusai VTE Cancer trial compared the recurrent VTE and major bleeding events in 1046 cancer patients with acute VTE receiving either LMWH for at least 5 days followed by oral edoxaban (60 mg once daily), or subcutaneous dalteparin (200 IU/kg once daily) for 1 month followed by dalteparin (150 IU/kg once daily), for at least 6 months and up to 12 months. Recurrent VTE events occurred in 41 patients (7.9%) in the edoxaban group (*n* = 522) and in 59 patients (11.3%) in the dalteparin group (*n* = 524) (RD, − 3.4%; 95% CI, − 7.0 to 0.2). Major bleeding events occurred in 36 patients (6.9%) in the edoxaban group and in 21 patients (4.0%) in the dalteparin group (RD, 2.9%; 95% CI, 0.1 to 5.6) [21]. The Hokusai VTE Cancer study then evaluated the composite of recurrent VTE or major bleeding events over 12 months, the primary outcome occurred in 19.4% patients receiving edoxaban (*n* = 477) and in 15.0% receiving dalteparin (*n* = 465) (RD, 4.4%; 95% CI, − 4.1 to 12.8%) [22]. In the SELECT-D:12 m trial, the cumulative VTE recurrence from 6 months through 12 months after VTE diagnosis was 14% with placebo (*n* = 46) and 4% with rivaroxaban (*n* = 46) (hazard ratio, 0.32; 95% CI, 0.06–1.58). The major and non-major CRB rates were 0 and 0% with placebo, and 5% (95% CI, 1–18) and 4% (95% CI, 1–17) with rivaroxaban [23].

A retrospective study reviewed 322 cancer patients from 6 months through 24 months after the diagnosis of cancer-associated VTE. Anticoagulation was continued in 222 patients (68.9%) after 6 months’ anticoagulant treatment. Patients who continued anticoagulation beyond 6 months mostly had advanced cancer or anticancer therapy compared with those who discontinued anticoagulation [24]. In a meta-analysis, long-term treatment of DOACs were associated with a significant decrease of recurrent VTE and similar bleeding risk compared to long-term LMWH and VKA [10].

## Treatment of catheter-related VTE in patients with Cancer

The evidence regarding the treatment of catheter-related VTE in cancer patients are scarce. A prospective study assessed the efficacy and safety of rivaroxaban in 70 cancer patients with upper extremity deep vein thrombosis due to CVC (CVC-UEDVT). After 12 weeks of follow-up, the preservation of CVC function was 100%. The recurrent VTE event was observed in one episode of fatal PE (1.43%) at 12 weeks. Nine patients (12.9%) experienced a total of eleven bleeding episodes [25].

## Treatment of recurrent Cancer-associated VTE

Poor adherence, temporary cessation, inadequate dosage, cancer progression, and heparin-induced thrombocytopenia (HIT) can lead to recurrent VTE in cancer patients [26]. A study investigated the management of recurrent VTE in 212 cancer patients with breakthrough VTE which was defined as the first objectively verified VTE event after the initiation of anticoagulant therapy. The anticoagulation regimen changes after the breakthrough VTE were: dose unchanged in 33%, dose increased in 31%, switched to another agent in 24%, and other management in 11%. After 3 months of follow-up, additional VTE recurrence was less common with LMWH than with VKA (HR, 0.28; 95% CI, 0.11–0.70) [27].

## Treatment of Cancer-associated VTE in special situations

Several special clinical situations deserve extra attention in the treatment of cancer-associated VTE. Although advanced age is a risk factor for bleeding, anticoagulation should be offered to elderly patients without contraindications [5]. However, for cancer patients with intracranial malignancy, thrombocytopenia, renal impairment, obesity and pregnancy, anticoagulation treatment should be adjusted as per the specific situation [2, 5].

### Intracranial malignancy

Patients with intracranial malignancy are at increased risk of thrombotic complications and intracranial hemorrhage (ICH) simultaneously. A recent meta-analysis reviewed seven studies to determine the difference of ICH risk between glioma patients who underwent anticoagulation for established VTE and those who did not. The OR for ICH in glioma patients with VTE who received therapeutic anticoagulation compared with those who did not was 3.66 (95% CI, 1.84–7.29; I^2^ = 31%) [28]. However, the presence of primary or metastatic intracranial malignancy being active or not is not an absolute contraindication to anticoagulation [5]. A retrospective comparative cohort study compared the cumulative incidence of ICH between the use of DOACs and LMWH for 12 months in 172 patients with brain tumors and VTE. In the primary brain tumor cohort (*n* = 67), the cumulative incidence of any ICH was 0% in the DOACs group and 36.8% (95% CI, 22.3–51.3%) in the LMWH group, respectively. In the brain metastases cohort (*n* = 105), the incidence of major ICH was11.1% (95% CI, 0.5–40.6%) vs 17.8% (95% CI,10.2–27.2%) in DOACs and LMWH groups, respectively [29].

### Thrombocytopenia

Thrombocytopenia does not reduce the risk of VTE. It is not uncommon for thrombocytopenic patients with cancer to have an indication for anticoagulant therapy. Since bleeding risk due to anticoagulation increase when platelets are less than 50 × 10^9^/L, management options may include no change, temporarily withholding anticoagulant treatment, reducing dosage, switching to other regimens, and platelet transfusion in this situation [30]. In a retrospective study, the conventional dose of dalteparin 200 units/kg once daily was adopted for patients with platelets more than 50 × 10^9^/L. For patients with thrombocytopenia (platelets of 25–50 × 10^9^ /L) (*n* = 75), the dosage of dalteparin was reduced to 100 units/kg once daily. For patients with platelets less than 25 × 10^9^/L (*n* = 77), dalteparin was withheld unless platelets recovered to exceed the 25 × 10^9^/L threshold. During a two-year observation, new or recurrent VTE was documented in 2.6, 0, and 2.2% of patients with platelet counts of< 25, 25–50, and > 50 × 10^9^/L, respectively. (*p* > 0.9 for all comparisons). Acute blood loss or major bleeding events were documented in 10.5, 12.5, and 15.6% of patients with platelet counts of< 25, 25–50, and > 50 × 10^9^/L, respectively (*p* > 0.9 for all comparisons) [31]. In the RIETE study, 166 (1.1%) had platelets less than 50 × 10^9^ /L (severe thrombocytopenia), 711 (4.6%) had platelets of 50–99 × 10^9^/L (mild thrombocytopenia) and 14,460 (94.3%) had platelets more than 100 × 10^9^/L (normal count). Most patients received LMWH at conventional dose for initial therapy, whereas 62% of those with severe thrombocytopenia received LMWH less than150 IU/kg/day, and 42% of them received LMWH less than 100 IU/kg/day. The mortality rate progressively decreased along with the increase of platelet counts (12, 9.4 and 3.3% at 10 days, 27, 18 and 9.4% at 30 days, respectively), whereas the major bleeding rates did not differ among three groups (1.2, 2.5 and 1.3% at 10 days, 2.4, 4.4 and 2.2% at 30 days, respectively) [32].

### Renal impairment

Bleeding risk is high in cancer patients with concurrent renal impairment which may lead to the accumulation of anticoagulant agents within the body [5]. In the CATCH trial, between patients with and without renal impairment, the recurrent VTE rates were 14 and 8% (RR 1.74; 95% CI 1.06, 2.85), whereas major bleeding rates were 6.1 and 2.0% (RR 2.98; 95%CI 1.29, 6.90) and mortality rates were 40 and 34%, respectively (RR 1.20; 95% CI 0.94, 1.53) [33]. In the CLOT trial, for cancer patients with renal impairment (creatinine clearance< 60 ml/min), less dalteparin-treated patients (2/74 [2.7%]) experienced more than one adjudicated symptomatic VTE recurrence compared with VKA-treated patients (15/88 [17.0%]; HR = 0.15 [95%CI 0.03–0.65]; *p* = 0.01). Bleeding rates were similar between two treatments (*p* = 0.470) [34].

### Obesity

Anticoagulation dose has been understudied in large or obese patients. In a retrospective study with 7602 cancer patients receiving thromboprophylaxis, body mass index (BMI) was found to be a significant predictor of VTE (OR = 1.094, 95% CI 1.021–1.172, *p* = 0.011). Cancer patients with high BMI may pose a risk of breaking through standard thromboprophylaxis dosage [35].

### Pregnancy

Pregnancy is another special situation in the treatment of cancer-associated VTE. The evidence regarding this situation is also scarce. A prospective study compared the maternal morbidity and mortality between 29 pregnant cancer patients with high-risk of VTE receiving parenteral thromboprophylaxis for 3 months and 23 ones with low-risk of VTE not receiving thromboprophylaxis. Among 29 patients receiving thromboprophylaxis, no one exhibited VTE, adverse effects of thromboprophylaxis or death after 3 months of thromboprophylaxis [36].

## Comparison among different therapeutic anticoagulant agents

There are a large number of studies with respect to the comparison of efficacy and safety between different frequently-used anticoagulant agents for established cancer-associated VTE. DOACs vs LMWH is the most frequent comparison, followed by DOACs vs VKA and LMWH vs VKA.

### DOACs vs LMWH

In recent years, with respect to DOACs vs LMWH, besides SELECT-D trial [37], six meta-analyses indicated that DOACs were more efficacious in the treatment of cancer-associated VTE with an increase of bleeding risk, compared with LMWH [11, 20, 38–41], whereas a retrospective study [7] and another five meta-analyses [10, 15, 16, 42, 43] indicated that DOACs were more efficacious with a comparable bleeding risk, compared with LMWH. In the ADAM VTE trial, major bleeding and VTE recurrence rates were both lower in cancer patients with VTE receiving oral apixaban than those receiving dalteparin [44]. The Caravaggio trial (apixaban) [14], Hokusai VTE Cancer trial (edoxaban) within 12 months [21] and over 12 months [22] reported that DOACs were noninferior to LMWH (dalteparin) with similar bleeding risk in the treatment of cancer-associated VTE, whereas two meta-analyses [45, 46] showed that DOACs were noninferior to LMWH with an increase of bleeding risk. In addition, the AMPLIFY trial indicated that DOAC (apixaban) was more efficacious than LMWH (enoxaparin) followed by VKA (warfarin) with less bleeding risk [47].

### DOACs vs VKA

With regard to DOACs vs VKA in recent years, one retrospective study [7] and two meta-analyses [10, 42] reported that DOACs were more efficacious in the treatment of cancer-associated VTE with similar bleeding risk compared to VKA, whereas another two meta-analyses [11, 38] indicated that DOACs were more efficacious with a decrease of bleeding risk, compared to VKA. Besides, the Hokusai-VTE trial reported that DOAC (edoxaban) was as effective as VKA (warfarin) for the treatment of patients with cancer-associated VTE with less bleeding risk [48].

### LMWH vs VKA

As for LMWH vs VKA, two meta-analyses [11, 20] and one retrospective study [34] reported that LMWH was more efficacious in the treatment of cancer-associated VTE with similar bleeding risk compared with VKA, whereas the CATCH trial [49] and one meta-analysis indicated that the therapeutic efficacy for cancer-associated VTE and bleeding risk were both similar between LMWH and VKA [38].

Anyway, no significant difference was found in all-cause mortality among cancer patients with established VTE treated with DOACs, LMWH and VKA to date [1, 2, 5]. Although the preference for oral anticoagulants over injection is moderately important, most patients do not think it is difficult, painful, or inconvenient to use LMWH regimen [1]. So far, LMWH is still the first choice for the treatment especially the initial treatment of cancer-associated VTE [1, 2, 5]. Dalteparin, edoxaban, and rivaroxaban have the highest priority in the selection of anticoagulants [1, 5]. The comparison among different randomized clinical trials regarding DOACs vs LMWH in the treatment of cancer-associated VTE is in Table 1.

**Table 1:** Comparison among different randomized clinical trials assessing the efficacy and safety of DOACs in the treatment of cancer-associated VTE

	SELECT-D [37]	Hokusai VTE-Cancer [22]	ADAM-VTE [44]	Caravaggio [14]
Trial design	Open-label, pilot	Open-label, noninferiority	Open-label, investigator-initiated	Open-label, controlled, investigator-initiated, noninferiority
Number of patients	406	942	300	1155
DOACs	Rivaroxaban (15 mg twice daily for 3 weeks, followed by 20 mg once daily for 2–6 months)	Edoxaban (dalteparin for at least 5 days, followed by edoxaban 60 mg once daily, for 12 months)	Apixaban (10 mg twice daily for the first week, followed by 5 mg twice daily for 6 months)	Apixaban (10 mg twice daily for the first week, followed by 5 mg twice daily for 6 months)
Comparators	LMWH (daltaparin 200 IU/kg once daily for the first month, followed by 150 IU/kg daily for 2–6 months)	LMWH (daltaparin 200 IU/kg once daily for the first month, followed by 150 IU/kg daily for 12 months)	LMWH (daltaparin 200 IU/kg once daily for the first month, followed by 150 IU/kg daily for 6 months)	LMWH (dalteparin 200 IU/kg once daily for the first for the first month, followed by 150 IU/kg once daily for 6 months)
Inclusion criteria	Patients with active cancer and symptomatic or incidental PE, or symptomatic lower extremity proximal DVT	Patients with active cancer and acute symptomatic or incidental proximal DVT and/or PE	Patients with active cancer and acute extremity DVT, PE, splanchnic or cerebral vein thrombosis	Patients with active cancer and acute symptomatic or incidental proximal DVT or PE
Cancer types	Solid and hematologic malignancies (other than basal-cell or squamous-cell skin carcinoma)	Gastrointestinal, lung, urogenital, breast, hematological, and gynecological cancer	Solid and hematologic malignancies	Cancers other than basal-cell or squamous-cell carcinoma of the skin, primary brain tumor, intracerebral metastases, or acute leukemia
Primary outcome measurements	VTE recurrence	The composite of VTE recurrence or major bleeding	Episode of major bleeding	VTE recurrence
Primary outcome results	Rivaroxaban 4% (95% CI, 2 to 9%); Dalteparin 11% (95% CI, 7 to 16%); (HR, 0.43; 95% CI, 0.19 to 0.99)	Edoxaban 19.4%; Dalteparin 15.0%; (RD, 4.4%; 95%CI, − 4.1 to 12.8%)	Apixaban 0%; Dalteparin 1.4%;(*P* = 0.138)	Apixaban 5.6%; Dalteparin 7.9%; (HR, 0.63; 95% CI, 0.37 to 1.07; P < 0.001 for noninferiority)

Note: *DOACs* Direct oral anticoagulants, *VTE* Venous thromboembolism, *LMWH* Low-molecular-weight heparin, *PE* Pulmonary embolism, *DVT* Deep venous thrombosis

## Guidelines

According to the latest guidelines, anticoagulation options recommended for treatment of cancer-associated VTE include one-agent regimens (e.g. LMWH or DOACs) (monotherapy) and regimens of more than one agent (e.g. LMWH+DOACs) (combination therapy) [1]. LMWH, UFH, fondaparinux, rivaroxaban or apixaban can be used for the initial 5 to 10 days of anticoagulation in cancer patients with acute diagnosed VTE in the absence of severe renal impairment. LMWH is preferred over others [1, 2, 5]. With respect to the usage frequency of LMWH, once daily is recommended unless a twice-daily regimen is necessary in the ITAC guidelines [2], whereas twice-daily dosing is considered to be more efficacious than once-daily dosing in the ASCO guidelines [5]. DOACs can be used in cancer patients without severe renal impairment, gastrointestinal or genitourinary malignancy or bleeding risk, previous small bowel surgery, or strong drug–drug interactions [1, 2, 5]. Thrombolysis may only be recommended to cancer patients with VTE without contraindications on a case-by-case basis, by clinicians experienced with parenteral or CDT [2], whereas the insertion of IVCF is usually offered to cancer patients with life-threatening VTE and absolute or relative contraindications to anticoagulant therapy, or recurrent VTE despite optimal anticoagulation [2, 5]. Incidental VTE should be treated in the same manner as symptomatic VTE, whereas the treatment of subsegmental PE or SPVT should be discussed on a case-by-case basis [5]. Unless there is contraindication, LMWH or DOACs should be used for a minimum of 6 months for established acute cancer-associated VTE [2, 5].

Anticoagulation beyond 6 months can be offered to patients with active cancer [5]. Termination or continuation of anticoagulation after 6 months’ treatment of cancer-associated VTE should be based on an intermittent individual assessment of the benefit–risk ratio, tolerability, patient preference, and cancer activity [2, 5].

For the treatment of symptomatic acute catheter-related VTE in cancer patients, anticoagulant treatment is recommended for a minimum of 3 months. LMWH is recommended over other anticoagulants in this setting. As long as the CVC is in place, anticoagulation is recommended in cancer patients with established catheter-related VTE [2].

For patients with recurrence of cancer-associated VTE after standard doses of anticoagulant therapy, treatment adherence, HIT, or mechanical compression due to malignancy progression should be reassessed [5]. Switching to an alternative anticoagulant regimen or increasing the dose of LMWH usually are the optional management [2, 5]. Three options may be considered: (1) for LMWH, increase the dose by 20–25% or switch to DOACs; (2) for DOACs, switch to LMWH; and (3) for VKA, switch to LMWH or DOACs [2]. An IVCF in addition to LMWH can be reserved for recurrent cancer-associated VTE as a last resort [2, 5].

LMWH or DOACs should be offered for patients with established VTE and primary or metastatic intracranial malignancy [2, 5]. For cancer patients with platelet count more than 50 × 10^9^/L, anticoagulation can be used for the treatment of established VTE without the evidence of bleeding. For patients with platelet count below 50 × 10^9^/L, treatment decisions as well as dosage should be deliberated with the extreme caution on a case-by-case basis, whereas platelet count below 20 × 10^9^/L is an absolute contraindication to anticoagulation [2, 5]. For cancer patients with severe renal failure, UFH followed by VKA or LMWH adjusted to anti-Xa level are recommended in this situation [2, 5]. In addition, an external compression device can be applied [2]. For cancer patients with a BMI greater than 40 kg/m^2^ or a weight greater than 120 kg, LMWH is likely preferred over DOACs. If DOACs are used, the monitoring of drug-specific peak and trough levels are advised [5]. For cancer patients with pregnancy, LMWH is recommended for the treatment of established VTE. VKA and DOACs should be avoided in this setting [2].

**Fig. 1:**
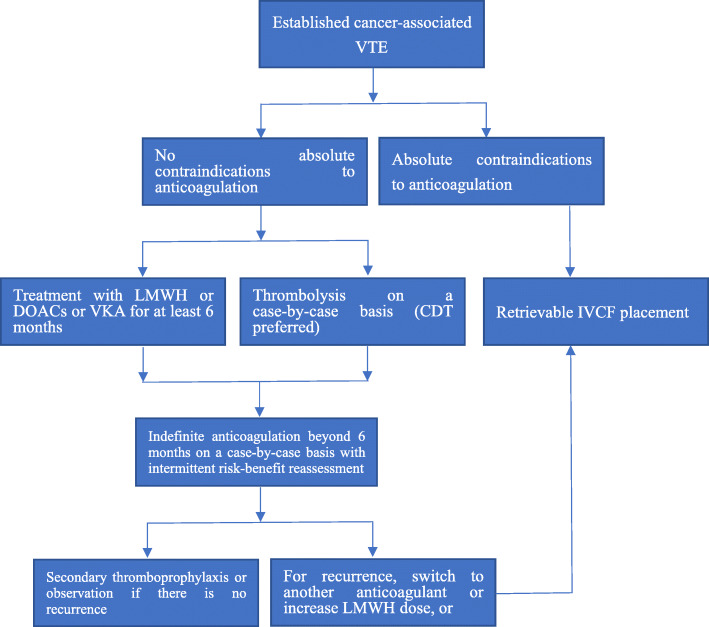
Currently Recommended Treatment Procedure of Cancer-associated VTE. Note: VTE: venous thromboembolism; LMWH: low-molecular-weight heparin; DOACs: direct oral anticoagulants;VKA: vitamin K antagonist; CDT: catheter directed thrombolysis; IVCF: inferior vena cava filter

The comparison of recommendations regarding treatment of established cancer-associated VTE between the latest ASCO [5] and ITAC guidelines [2] are in Table 2. The comparison of contraindications to therapeutic anticoagulant therapy of cancer-associated VTE between the latest National Comprehensive Cancer Network (NCCN) [1] and ASCO [5] guidelines are in Table 3. The comparison of recommendations regarding anticoagulation of cancer-associated VTE in special situations between the latest ASCO [5] and ITAC [2] guidelines are in Table 4. Based on the latest guidelines [1, 2, 5], the currently recommended treatment procedure of cancer-associated VTE is demonstrated in Fig. 1.

**Table 2:** Comparison of recommendations regarding treatment of established cancer-associated VTE between the latest ASCO [5] and ITAC [2] guidelines

	ASCO	ITAC
Duration	Initial treatment (typically first 5–10 days) + long-term treatment (6 months) + treatment after 6 months	Initial treatment (typically first 5–10 days) + early maintenance (6 months) + long-term treatment (6 months later)
Treatment within 6 months		
1. Agents	LMWH (twice-daily preferred) or UFH or fondaparinux or DOACs is recommended when CrCl is ≥30 mL/min	LMWH (once-daily preferred) or DOACs or UFH or fondaparinux is recommended when CrCl is ≥30 mL/min
2. Thrombolysis	Not mentioned	Only for patients without contraindications on a case-by-case basis (CDT considered)
3. IVCF	Only for patients with life-threatening VTE and absolute contraindications to anticoagulation (retrievable filter preferred)	May be considered for patients with contraindications to anticoagulation
Treatment after 6 months	The use of LMWH or DOACs or VKA should be offered to active cancer patients with intermittent risk-benefit reassessment	The use of LMWH or DOACs or VKA should be based on individual evaluation of the benefit–risk, tolerability, drug availability, patient preference, and cancer activity
Catheter-related VTE	Not mentioned	LMWH is recommended for a minimum of 3 months and as long as the CVC is in place
Recurrent VTE		
1. Anticoagulant regimen	Switching to an alternative anticoagulant regimen or increasing the dose of LMWH may be considered	For LMWH, increase the dose by 20–25% or switch to DOACs; for DOACs, switch to LMWH; for VKA, switch to LMWH or DOACs
2. IVCF	May be offered to patients with progression of thrombosis despite optimal anticoagulation as a last resort (retrievable filter preferred)	May be considered for patients with recurrent pulmonary embolism despite optimal anticoagulation
Incidental VTE	Treated in the same manner as symptomatic VTE	Not mentioned
Subsegmental PE or SPVT	Treatment should be offered on a case-by-case basis	Not mentioned

Note: *VTE* Venous thromboembolism, *ASCO* American Society of Clinical Oncology, *ITAC* International Initiative on Thrombosis and Cancer, *LMWH* Low-molecular-weight heparin, *UFH* Unfractionated heparin, *DOACs* Direct oral anticoagulants, *CrCl* Creatinine clearance, *CDT* Catheter directed thrombolysis, *IVCF* Inferior vena cava filter, *VKA* Vitamin K antagonist, *CVC* Central venous catheter, *PE* Pulmonary embolism, *SPVT* Splanchnic vein thrombosis

**Table 3:** Comparison of Contraindications to Therapeutic Anticoagulant Therapy of Cancer-associated VTE Between the Latest NCCN [1] and ASCO [5] Guidelines

	NCCN	ASCO
Absolute contraindications	1. Recent/acute HIT for LMWH or UFH 2. Severe renal dysfunction (CrCl < 30 mL/min) for fondaparinux or DOACs 3. Active/clinically significant liver disease for DOACs 4. Concomitant use of strong dual inhibitors/inducers of CYP3A4 and P-glycoprotein for DOACs 5. Concomitant use of inducers/inhibitors of P-glycoprotein for DOACs	1. Active major, serious, or potentially life-threatening bleeding 2. Severe, uncontrolled malignant hypertension 3. Severe, uncompensated coagulopathy 4. Severe platelet dysfunction or inherited bleeding disorder 5. Persistent, severe thrombocytopenia (<20 × 10^9^/L) 6. High-risk invasive procedure in a critical site 7. Concurrent use of potent P-glycoprotein or CYP3A4 inhibitors or inducers for DOACs
Relative contraindications	1. Severe renal dysfunction (CrCl < 30 mL/min) for LMWH 2. Past history of HIT for LMWH or UFH 3. Moderate renal insufficiency (CrCl 30–50 mL/min), weight < 50 kg, or age > 75y for fondaparinux 4. Concomitant use of inhibitors/inducers of CYP2C9, 1A2, or 3A4 for VKA 5. Urinary or gastrointestinal tract lesions for DOACs 6. Compromised renal or liver function for DOACs 7. Patients receiving nephrotoxic or hepatotoxic chemotherapy for DOACs 8. Drug-drug interactions for DOACs	1. Intracranial or spinal lesion at high risk for bleeding 2. Active gastrointestinal ulceration at high risk of bleeding 3. Active but non–life-threatening bleeding 4. Intracranial or CNS bleeding within past 4 weeks 5. Recent high-risk surgery or bleeding event 6. Persistent thrombocytopenia (<50 × 10^9^/L)

Note: *VTE* Venous thromboembolism, *NCCN* National Comprehensive Cancer Network, *ASCO* American Society of Clinical Oncology, *HIT* Heparin-induced thrombocytopenia, *LMWH* Low-molecular-weight heparin, *UFH* Unfractionated heparin, *CrCl* Creatinine clearance, *DOACs* Direct oral anticoagulants, *CYP* Cytochrome P450, *CNS* Central nervous system, *VKA* Vitamin K antagonist

**Table 4:** Comparison of Recommendations Regarding Anticoagulation of Cancer-associated VTE in Special Situations Between the Latest ASCO [5] and ITAC [2] Guidelines

	ASCO	ITAC
Intracranial Malignancy	DOACs or LMWH should be offered to patients with established VTE and primary or metastatic CNS malignancies	LMWH or DOACs should be recommended for patients with established VTE and brain tumor or cancer patients undergoing neurosurgery
Thrombocytopenia	Anticoagulation is absolutely contraindicated when platelet count is persistently below 20 × 10^9^/L, and relatively contraindicated when platelet count is persistently below 50 × 10^9^/L	For established VTE, full doses of anticoagulant can be used when platelet count is>50 × 10^9^/L and should be deliberated case-by-case when platelet count is≤50 × 10^9^/L; prophylactic anticoagulation can be used when platelet count is>80 × 10^9^/L
Renal impairment	For moderate to severe renal impairment, LMWH adjusted to anti-Xa level or UFH followed by VKA are recommended	When CrCl is < 30 mL/min, UFH followed by VKA or LMWH adjusted to anti-Xa level are recommended; an external compression device can be applied
Obesity	For obese cancer patients (BMI>40 kg/m^2^ or a weight>120 kg), LMWH is preferred over DOACs; the monitoring of drug-specific peak and trough levels are advised if DOACs used	A higher dose of LMWH should be offered for obese cancer patients undergoing surgery
Pregnancy	Not mentioned	LMWH is recommended; VKA and DOACs should be avoided

Note: *VTE* Venous thromboembolism, *ASCO* American Society of Clinical Oncology, *ITAC* International Initiative on Thrombosis and Cancer, *DOACs* Direct oral anticoagulants, *LMWH* Low-molecular-weight heparin, *CNS* Central nervous system, *CrCl* Creatinine clearance, *UFH* Unfractionated heparin, *VKA* Vitamin K antagonist, *BMI* Body mass index

## Treatment of Cancer-associated VTE with COVID-19

Unfortunately, we are now living in the era of COVID-19. Thrombosis is one of the multiple potential mechanisms for cardiac injury due to COVID-19 [50], putting cancer patients who are already at a high risk of VTE at higher VTE risk. Nevertheless, in a retrospective cohort analyses of 398 consecutive patients hospitalized with COVID-19, the cumulative incidence of thrombotic events was 18.2% (95% CI, 10.2 to 27.9%) in non-cancer cohort (*n* = 353) and 14.2% (95% CI, 4.7 to 28.7%) in the active cancer cohort (*n* = 45) at day 28. The cumulative incidence of major and fatal bleeding was 20.8% (95% CI, 12.1 to 31.0%) in the non-cancer group and 19.5% (95% CI, 5.5 to 39.8%) in the cancer cohort at day 28 [51]. Therefore, applying the current guidelines to cancer patients with COVID-19 is challenging due to their high risk for both thrombosis and bleeding. To date, at a guidance level, parenteral anticoagulation (e.g. LMWH) is preferred over oral anticoagulation in the treatment of established VTE in cancer patients with COVID-19 [52].

## Conclusions

Cancer-associated venous thromboembolism is a concerning issue that increases both morbidity and mortality for patients with cancer. For established cancer-associated VTE including the incidental or asymptomatic one, the decision of treatment should be made based on the risk-benefit ratio, whereas the selection among anticoagulants should be made based on anticoagulant efficacy, bleeding risk assessment, renal or hepatic function, drug-drug interactions, clinical setting, convenience of use, monitoring, FDA approval, cost, drug availability and patient preference. In addition to LMWH, DOACs have shown a predominant role in the treatment of cancer-associated VTE, despite its adverse effects. To date, LMWH and DOACs are most highly recommended anticoagulants for the treatment of cancer-associated VTE. A minimum of 6 months of treatment should be offered to patients with cancer-associated VTE, whereas the continuation or discontinuation of treatment should be made on a case-by-case basis with intermittent assessment of risk-benefit ratio of anticoagulation. For recurrent cancer-associated VTE, the original anticoagulant regimen should be replaced or a retrievable IVCF can be applied. However, IVCF may only be regarded as a last resort in the case of absolute contraindications to anticoagulation or recurrence of cancer-associated VTE. The future direction of treatment of cancer-associated VTE should be focused on how to reduce its recurrence as well as all-cause mortality without bringing much risk, adverse events or discomfort to patients.

## Data Availability

Not applicable.
